# Consumption of Foods Derived from Subsidized Crops Remains Associated with Cardiometabolic Risk: An Update on the Evidence Using the National Health and Nutrition Examination Survey 2009–2014

**DOI:** 10.3390/nu12113244

**Published:** 2020-10-23

**Authors:** Whitney L. Do, Kai M. Bullard, Aryeh D. Stein, Mohammed K. Ali, K. M. Venkat Narayan, Karen R. Siegel

**Affiliations:** 1 Department of Global Health, Rollins School of Public Health, Emory University and Nutrition and Health Sciences Program, Laney Graduate School, Emory University, Atlanta, GA 30022, USA; 2 Division of Diabetes Translation, Centers for Disease Control and Prevention, Atlanta, GA 30341, USA; hjo1@cdc.gov (K.M.B.); yuo0@cdc.gov (K.R.S.); 3 Department of Global Health, Rollins School of Public Health, Emory University, Atlanta, GA 30022, USA; aryeh.stein@emory.edu (A.D.S.); mkali@emory.edu (M.K.A.); knaraya@emory.edu (K.M.V.N.)

**Keywords:** farm subsidies, cardiometabolic disease, food policy, obesity

## Abstract

In this study, we examined the associations between the consumption of foods derived from crops subsidized under the 2008 United States (US) Farm Bill and cardiometabolic risk factors and whether the magnitude of these associations has changed since the 2002 US Farm Bill. Four federal databases were used to estimate daily consumption of the top seven subsidized commodities (corn, soybeans, wheat, rice, sorghum, dairy, and livestock) and to calculate a subsidy score (0–1 scale) for Americans’ daily dietary intake during 2009–2014, with a higher score indicative of a higher proportion of the diet derived from subsidized commodities. The cardiometabolic risk factors included obesity, abdominal adiposity, hypertension, dyslipidemia, and dysglycemia. Linear and logistic regression models were adjusted for age, sex, race/ethnicity, the poverty–income ratio, the smoking status, educational attainment, physical activity, and daily calorie intake. During 2009–2014, adults with the highest subsidy score had higher probabilities of obesity, abdominal adiposity, and dysglycemia compared to the lowest subsidy score. After the 2002 Farm Bill (measured using data from 2001–2006), the subsidy score decreased from 56% to 50% and associations between consuming a highly-subsidized diet and dysglycemia did not change (*p* = 0.54), whereas associations with obesity (*p* = 0.004) and abdominal adiposity (*p* = 0.002) significantly attenuated by more than half. The proportion of calories derived from subsidized food commodities continues to be associated with adverse cardiometabolic risk factors, though the relationship with obesity and abdominal adiposity has weakened in recent years.

## 1. Introduction

During the past two decades, adult obesity rates have risen by 30% in the United States (US); currently, two in three individuals are overweight or obese [1]. With the rise in rates of obesity, there has been a concurrent rise in associated cardiometabolic diseases, such as cardiovascular disease and type 2 diabetes. Obese individuals have a 67–85% increased risk of cardiovascular disease [2] and 37% increased risk of type 2 diabetes [3] compared with those who are not obese. This rise in the obesity rate can largely be attributed to changes in dietary and physical activity patterns [4]. Agricultural subsidies from the US Farm Bill, which primarily support the production of corn, soybeans, wheat, rice, sorghum, dairy, and livestock feed, may be playing a role in unhealthy food consumption patterns and, subsequently, in the development of obesity and diet-related chronic diseases [5]. For example, corn represents one of the most widely produced and federally subsidized crops in the US, with the majority of its product ending up in animal and livestock feed or heavily processed foods as a high fructose corn syrup, including sugar-sweetened beverages (SSBs), cereals, and alcohol [6]. Extensive research has shown that the consumption of these processed foods is associated with obesity [7,8,9,10]. In response to the high consumption of SSBs and heavily processed foods, as well as the obesity epidemic [11], local policymakers have considered taxing these foods [12] at the point of purchase, even though these products may have been derived from federally subsidized crops. This paradox reinforces the need to examine the relationship between the consumption of foods derived, in part, from subsidies and cardiometabolic diseases.

Previously, using data from the 2001–2006 National Health and Nutrition Examination Surveys (NHANES) linked with other federal databases, we developed a scoring algorithm to estimate the proportion of foods derived from subsidized commodities in an individual’s diet [13]. We then used this scoring algorithm to examine the relationship between an individual’s consumption of foods derived from subsidized crops and their cardiometabolic risk [13,14]. Aligning with the 2002 US Farm Bill, we found that a higher consumption of foods derived from the most heavily subsidized commodities was associated with a higher probability of adverse cardiometabolic risk factors, as measured by the body mass index (BMI), abdominal adiposity, C-reactive protein level, lipid level, and hemoglobin A1c (HbA1c) in adults aged 18–64 years [13]. Although novel, this assessment used data that were more than 10 years old and this relationship may have changed in subsequent Farm Bill cycles due to shifts in Farm Bill policies [15], as well as health and demographic shifts during this period, including the 2008 recession or public health campaigns focused on healthy eating [16,17,18]. In this analysis, we replicated the previous study using more recent data from NHANES 2009–2014, in alignment with the 2008 Farm Bill, released for the period of 2008–2012 and renewed until 31 December, 2013 [19]. We assessed whether the consumption of subsidized foods has changed over time, whether the association between the consumption of subsidized foods and cardiometabolic risk factors has persisted in recent years, and the extent to which the magnitude of the association may have changed from the 2002 US Farm Bill (NHANES 2001–2006, T1) to the 2008 US Farm Bill (2009–2014, T2).

## 2. Materials and Methods

### 2.1. Study Population

NHANES is a cross-sectional survey of a representative sample of noninstitutionalized civilians among the US population. We pooled data from the 2009–2010, 2011–2012, and 2013–2014 NHANES cycles. For comparison to T1, we pooled data from 2001–2002, 2003–2004, and 2005–2006 NHANES cycles. The study population included non-pregnant adults aged 18 to 64 years who provided dietary intake data and were consuming between 800 and 5000 kcals per day, outside of which has been previously reported as extreme levels of intake [13,20]. This yielded a final sample of 12,039 individuals from T2 and 10,309 individuals from T1.

### 2.2. Subsidy Score

The subsidy score estimation methodologies have been described in detail [14]. NHANES dietary intake data were derived from the one-day 24-hour recall using the USDA Automated Multiple-Pass Method, which employs both an unstructured and structured approach to probe the participant on their dietary intake over the past 24 hours [21]. We used data from the NHANES [22,23,24], including What We Eat in America (WWEIA) [25,26,27], the Food Pattern Equivalency Database (FPED) [28,29,30], Food Intakes Converted to Retail Commodities (FICRCD) [31], and the National Nutrient Database for Standard Reference [32], to estimate the amount of food in grams derived from corn, soybeans, wheat, rice, sorghum, dairy, and livestock in each individual’s daily food consumption. Specifically, these four databases estimated the amount of subsidized commodity in grams per 100 gram of each food item in NHANES. The FICRCD provided estimates for whole corn, corn flour/starch, soyabean meal (feed), grain sorghum (feed), wheat, rice, dairy (milk, yogurt, cheese, and butter) and meat (beef, pork, and chicken). The FPED provided estimates for soya products and added sweeteners (corn sweetener). Finally, WWEIA provided estimates for soyabean oil, farm-raised fish, and sorghum syrup [14]. The subsidy score ranged from 0.00 to 1.00, with 0.00 indicating 0% of the total energy intake coming from subsidized commodities and 1.00 indicating 100% of the total energy intake coming from subsidized commodities. The scores were categorized into quartiles identified within the sample. For T2, quartiles were defined as follows: Q1 is 0.00–0.41; Q2 is 0.42–0.53; Q3 is 0.54–0.63; and Q4 is 0.64–1.00. We also performed a sensitivity analysis using the quartiles from T1 (Q1 is 0.00–0.47, Q2 is 0.48–0.57, Q3 is 0.58–0.65, and Q4 is 0.66–1.00).

### 2.3. Cardiometabolic Risk Measures

Cardiometabolic factors were assessed as continuous measures of BMI (kg/m^2^) calculated from the measured height and body weight, ratio of waist circumference to height, systolic blood pressure (SBP, mmHg), diastolic blood pressure (DBP, mmHg), non-HDL cholesterol (mg/dL), and HbA1c (%). Methods applied for the measurement of these variables have previously been described in detail [33,34].

We also examined dichotomized cardiometabolic risk factors: Obesity (BMI ≥ 30 kg/m^2^); abdominal adiposity (measured by the ratio of waist circumference to height ≥ 0.59, which has been shown to to be a signficant risk factor for cardiovascular disease [35,36,37]); hypertension (SBP ≥ 140 mmHg or DBP ≥ 90 mmHg, self-report of a health care provider hypertension diagnosis or the use of hypertensive medications); hyperlipidemia (non-HDL cholesterol ≥ 160 mg/dL, self-report of a health care provider hyperlipidemia diagnosis or the use of cholesterol medications); and dysglycemia (HbA1c ≥ 5.7% or self-report of a health care provider diabetes diagnosis) [38].

### 2.4. Covariates

Additional variables that were examined for stratified analyses and adjustment in models included age (years), race/ethnicity (non-Hispanic white, non-Hispanic black, Mexican American, and other), educational attainment (less than high school, high school graduate, at least some college, and college graduate or higher), the poverty–income ratio (PIR), the smoking status (current, past, and never smoker), participation in moderate/vigorous physical activity, and total daily caloric intake. Physical activity was dichotomized and defined by engagement in at least 10 min of leisure-time moderate or vigorous physical activity daily during a typical week (yes or no). The PIR is the ratio of a family’s income to the federal poverty level, and was categorized according to eligibility for food assistance programs: <130% of the poverty level (corresponding to eligibility for the Supplemental Nutrition Assistance Program and free school lunches); from 130% to <185% of the poverty level (eligible for the Special Supplemental Nutrition Program for Women, Infants and Children); and ≥185% of the poverty level (ineligible for federal food assistance programs).

### 2.5. Statistical Analysis

All statistical analyses were conducted in SAS, version 9.4 and SAS-callable SUDAAN, version 10.0. The weighted quartile of the subsidy score was defined by the study population from the dietary weights. We analyzed population characteristic proportions and means with the standard error (SE) across quartiles of the subsidy score. Differences in descriptive characteristics by quartiles of the subsidy score were examined using chi-square tests for categorical variables and ANOVA for continuous variables. We used linear regression to examine the relationship between subsidy score quartiles or continuous subsidy score with continuous cardiometabolic risk variables. We used multivariable logistic regression to examine the prevalence ratio (PR) of each dichotomized cardiometabolic risk measure across quartiles. All models were examined for interactions or adjusted for age, sex, race/ethnicity, educational attainment, PIR, the smoking status, moderate to vigorous physical activity, and total daily caloric intake.

To test for interactions between T1 and T2 and the subsidy score, we used multivariable logistic regression, including an interaction term for the time period and the subsidy score quartile, adjusting for the same covariates previously listed. To examine the difference in the overall subsidy score and the contribution of the individual food components to the subsidy score between the two time periods, linear regression models were examined, regressing the time period on individual subsidy score components and adjusting for the same covariates stated before. Statistical significance for all of the above analyses was set at *p* < 0.05.

## 3. Results

Table 1 shows the selected characteristics of US adults aged 18–64 years during T2. The mean age was 40.9 (SE: 0.29) years and 49.5% (SE: 0.48%) were male. Overall, 50.3% (SE = 0.003, 25%: 38.8, 75%: 62.0) of the total daily calories consumed were derived from subsidized commodities (Supplemental Figure S1). The consumption of subsidized commodities differed significantly across the categories of age, educational attainment, PIR, and leisure-time physical activity. Overall, those who reported consuming a higher proportion of subsidized foods in their dietary recalls were younger (18–24 years), poorer (<130% and 130% to <185% poverty level), less educated, consumed fewer daily calories, and engaged in less physical activity (all *p* < 0.0001), than those who consumed diets with a lower proportion of subsidized commodities.

There were small but significant associations between the subsidy score and mean levels of BMI, ratio of waist circumference to height, non-HDL cholesterol, and HbA1c, adjusting for covariates (Table 2). Each ten percentage point higher subsidy score was associated with a 0.23 kg/m^2^ higher BMI (95% CI: 0.13, 0.33; *p* < 0.0001, Table 3, Supplementary Materials Figure S2A), 0.003 higher waist to height ratio (95% CI: 0.002, 0.004; *p* < 0.0001, Table 3, Supplementary Materials Figure S2B), 1.11 mg/dL higher non-HDL cholesterol (95% CI: 0.61, 1.62; *p* < 0.0001, Table 3, Supplementary Materials Figure S2C), and 0.02% higher HbA1c (95% CI: 0.01, 0.03; *p* < 0.0001, Table 3, Supplementary Materials Figure S2D).

**Figure 1:**
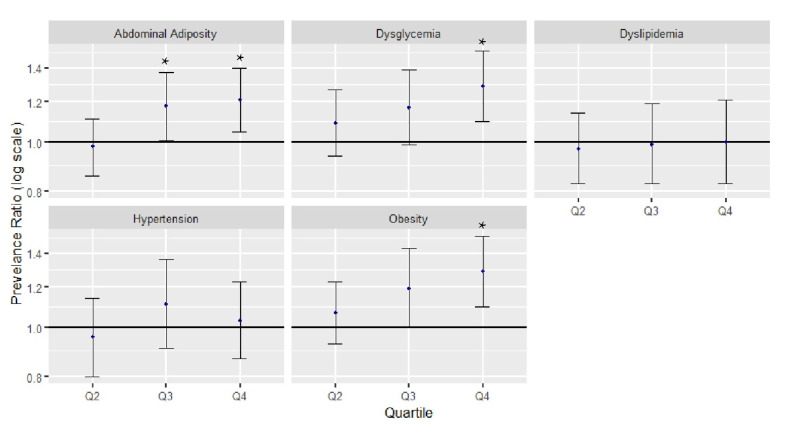
Adjusted prevalence ratio of cardiometabolic risk factors by subsidy score quartile in the National Health and Nutrition Examination Survey from 2009–2014. Subsidy score quartiles were defined as follows: Q1 is 0.00–0.41; Q2 is 0.42–0.53; Q3 is 0.54–0.63; and Q4 is 0.64–1.00. Q1 was used as a comparison group. Abdominal adiposity was defined as a ratio of waist circumference to height of at least 0.59. Dyslipidemia was defined as diagnosed (self-reported) or undiagnosed (no self-reported diagnosis and non-HDL cholesterol level ≥ 160 mg/dL) or currently taking anticholesterolemia medication. Dysglycemia was defined as a self-reported diabetes diagnosis or hemoglobin A1c level of at least 5.7%. Hypertension was defined as diagnosed (self-reported) or undiagnosed (no self-reported diagnosis and systolic blood pressure ≥ 140 mm Hg or diastolic blood pressure ≥ 90 mm Hg) or currently taking antihypertensive medication. Obesity was defined as a body mass index of at least 30 kg/m^2^. Individuals with missing data were excluded from the models. Model adjusted for sex, age, race/ethnicity, the highest education level, the poverty–income ratio, the smoking status, participation in moderate to vigorous physical activity, and total daily caloric intake. * *p* < 0.05.

When examining dichotomized cardiometabolic risk factors by subsidy score quartiles, those in the highest quartile (Q4) had a 29% higher likelihood of obesity (PR 1.29, 95% CI: 1.10, 1.51), a 21% higher likelihood of abdominal adiposity (PR 1.21, 95% CI: 1.05, 1.40), and a 29% higher likelihood of dysglycemia (PR 1.29, 95% CI: 1.10, 1.51) compared to those in the lowest quartile (Q1) when adjusting for covariates (Figure 1). Race/ethnicity interacted with the subsidy score in models examining abdominal adiposity (*p* = 0.0021). No effects across quartiles were found in non-Hispanic black individuals and Mexican Americans. In comparison, non-Hispanic whites and other races both showed significant associations between the subsidy score and abdominal adiposity, such that non-Hispanic whites and other races in Q4 had a 12% and 20% higher likelihood of abdominal adiposity compared to Q1, respectively (non-Hispanic whites PR 1.12, 95% CI: 1.00, 1.25; other races PR 1.20, 95% CI: 1.02, 1.41). Hypertension and dyslipidemia were not statistically significantly associated with the intake of subsidized commodities. As an aim of this study was to examine changes in the associations between cardiometabolic disease and the consumption of subsidized foods from the prior study, we conducted a sensitivity analysis examining these associations using the quartiles derived from the T1 (NHANES 2001–2006). When examining the associations using weighted quartiles defined in the previous analysis from T1, the associations remained consistent (Supplementary Materials Table S1 and S2).

**Figure 2:**
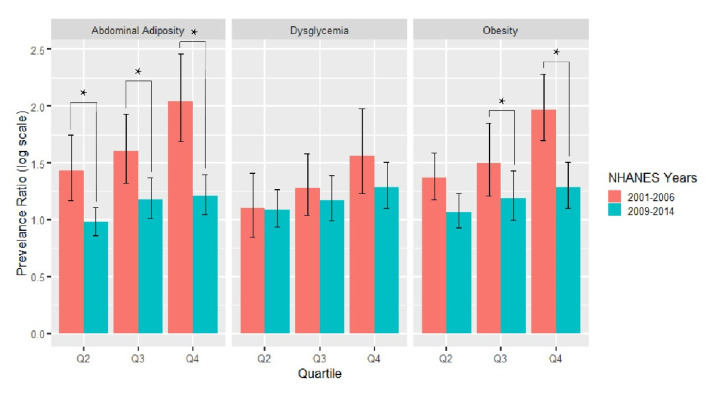
Adjusted prevalence ratio of cardiometabolic risk factors by subsidy score quartile in the National Health and Nutrition Examination Survey from 2009–2014 compared to the National Health and Nutrition Examination Survey 2001–2006. Subsidy score quartiles were defined as follows: Q1 is 0.00–0.41; Q2 is 0.42–0.53; Q3 is 0.54–0.63; and Q4 is 0.64–1.00. Obesity was defined as a body mass index of at least 30 kg/m^2^. Abdominal adiposity was defined as a ratio of waist circumference to height of at least 0.59. Dysglycemia was defined as a self-reported diabetes diagnosis or hemoglobin A1c level of at least 5.7%. Individuals with missing data were excluded from the models. Model adjusted for sex, age race/ethnicity, the highest education level, the poverty–income ratio, the smoking status, participation in moderate to vigorous physical activity, and total daily caloric intake. Difference in the prevalence ratio between time periods was compared using interaction with the time period; * *p* < 0.05.

**Figure 3:**
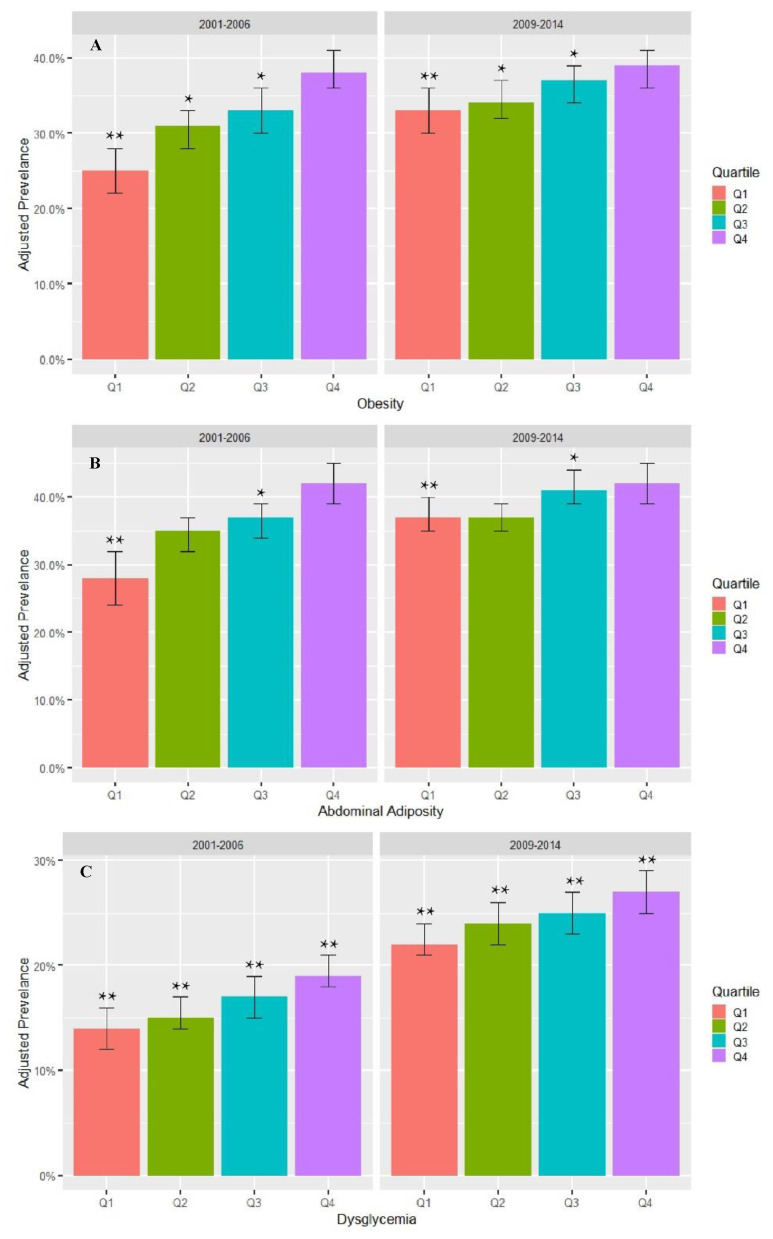
Adjusted prevalence of obesity (**A**), abdominal adiposity (**B**), and dysglycemia (**C**) by subsidy score quartile in the National Health and Nutrition Examination Survey between 2001–2006 and 2009–2014. Subsidy score quartiles were defined as follows: Q1 is 0.00–0.41; Q2 is 0.42–0.53; Q3 is 0.54–0.63; and Q4 is 0.64–1.00. Obesity was defined as a body mass index of at least 30 kg/m^2^. Abdominal adiposity was defined as a ratio of waist circumference to height of at least 0.59. Dysglycemia was defined as a self-reported diabetes diagnosis or hemoglobin A1c level of at least 5.7%. Individuals with missing data were excluded from the models. Model adjusted for sex, age, race/ethnicity, the highest education level, the poverty–income ratio, the smoking status, participation in moderate to vigorous physical activity, and total daily caloric intake. Significant differences in the adjusted prevalence within a quartile between the two time periods are denoted by * *p* < 0.05 and ** *p* < 0.0001.

Compared to T1, the consumption of subsidized food commodities during T2 decreased by 6 percentage points (*p* < 0.001), from 56.2% to 50.3%. We examined whether the relationship between the consumption of subsidized foods and cardiometabolic risk factors changed from T1 to T2. The association between dysglycemia and the subsidy score quartile was of a similar magnitude across the two time periods (p_interaction_ = 0.54). The associations between the consumption of subsidized foods and obesity and abdominal adiposity were attenuated from T1 to T2 (p_interaction_ = 0.004 and 0.002, respectively), even though they remained significantly associated with the subsidy score. Between the two periods, the association for obesity when comparing the highest (Q4) to the lowest consumers (Q1) of subsidized food commodities was 49% (PR_interaction_ = 1.49, 95% CI: 1.20, 1.84) higher in T1 compared to T2 (Figure 2). Similarly, the association for abdominal adiposity when comparing the highest (Q4) to the lowest consumers (Q1) of subsidized food commodities was 61% (PR_interaction_ = 1.61, 95% CI: 1.27, 2.04) higher in T1 compared to T2 (Figure 2). We additionally examined differences in the adjusted prevalence of each cardiometabolic risk factor by subsidy score quartile between the two time periods. While the association with the subsidy score may have attenuated, the adjusted prevalence of cardiometabolic risk increased between the two time periods within Q1 for all cardiometabolic factors, Q2 for obesity and dysglycemia, Q3 for all cardiometabolic factors, and Q4 for dysglycemia (all *p* < 0.05; Figure 3).

We also examined the changes in components of the subsidy score between the two time periods, in order to identify what may have accounted for the reduction in the subsidy score. Among components of the score, the mean subsidy score contribution decreased by 12% in corn sweetener (*p* < 0.0001), 14% in dairy (*p* < 0.0001), 10% in grains (*p* < 0.0001), 14% in soy (*p* = 0.002), and 51% in eggs (*p* < 0.0001) in T2 compared to T1. Only meat (*p* = 0.21) and corn-fed fish (*p* = 0.15) were not reduced (Table 4).

## 4. Discussion

We found that a higher consumption of subsidized commodities was associated with a higher prevalence of obesity, abdominal adiposity, and dysglycemia during 2009–2014. When cardiometabolic risk factors were examined continuously, a higher consumption of subsidized commodities was positively associated with the BMI, ratio of waist circumference to height, non-HDL cholesterol, and HbA1c. We found no significant associations between the subsidy score and hypertension.

Our findings corroborate those from our previous study examining these associations during 2001–2006 (T1) [13]. However, some results were significantly attenuated compared to the previous years. Overall, the percentage of calories derived from subsidized food commodities decreased from 56.2% of calories in T1 to 50.3% of calories in T2. The association between the subsidy score and dysglycemia did not change. The PRs comparing consumers with the highest subsidy score to the lowest subsidy score for obesity and abdominal adiposity were 49% and 61% higher during T1, respectively, suggesting a weaker relationship in recent years. However, the prevalence of obesity, abdominal adiposity, and dysglycemia significantly increased over this period. Therefore, the data from T2 had a lower distribution of healthy phenotypes across all the quartiles, which might explain the attenuation of this relationship.

We also examined changes in the specific food commodities making up the subsidy score. We identified a reduced intake of several components of the subsidy score, including corn sweetener, dairy, eggs, grains, and soy, whereas the intake of meat and corn-fed fish was not reduced. These results partially align with a study by Rehm et al. examining dietary patterns during this time period, which found significant reductions in milk and sugar-sweetened beverages (a major product of corn sweeteners), a stable intake of meat, and an increased intake of fish during 1999–2012 [39].

In this study, we were able to replicate the previous associations found in another NHANES population, reaffirming the associations between cardiometabolic disease and the intake of foods derived from subsidies. Changes in association may be the product of changes to the Farm Bill between the two cycles. While many of the provisions remained the same, adjustments to payment levels of commodities and a new average crop revenue election program were introduced in 2007 [15]. More recently, the 2014 Farm Bill made significant changes, with the elimination of direct payments for program crops and cuts to provisions for subsidies, and thus could be a useful comparator to previous cycles [40]. Future research efforts could evaluate how changes to the 2014 Farm Bill may have influenced these associations. Our study also examined data from six years following the 2008 recession, which may have influenced our findings. A few studies have found a significant effect of the recession on the overall diet quality and food security [16,17,18]. Though we adjusted for PIR (and found no differences when stratifying by PIR), other peripheral effects from the 2008 recession, including significant effects on mental health in marginalized populations [18], may have accounted for changes in the magnitude of association as anxiety and depressive symptoms can lead to both shifts in diet quality [41] and chronic disease risk factors [42].

Our findings also show that while cardiometabolic disease risk factors, such as obesity, abdominal adiposity, and dysglycemia, increased, there was a reduction in the intake of foods derived from subsidized commodities. This may be suggestive of improvements in dietary patterns overall, which is in alignment with a study by Wang et al. examining the change in diet quality measured by the Alternative Healthy Eating Index (AHEI)–2010 during 1999–2010. They found steady improvements in the AHEI-2010 score during this time period [43]. Any improvements in diet quality may be the product of public health campaigns focused on improving diets or shifts in dietary trends. For example, the *Let’s Move* campaign started by First Lady Michelle Obama in 2010 may have influenced adult dietary patterns given the increased media attention on healthy eating and encouragement to follow USDA’s MyPlate guidelines [44]. Additionally, policies from the *Let’s Move* campaign, including the Healthy Food Financing Initiative, that focused on improving food access in low-income communities, may have led to improved metrics of diet quality (such as those seen in our study), where the effects on cardiometabolic health would likely be latent [45].

The subsidy score was also a less substantial predictor of obesity and abdominal adiposity in recent years, suggesting that other unmeasured covariates may be contributing to the rise in obesity and abdominal adiposity. For example, some changes in sedentary behavior have been noted during this period, with a rise in leisure-time computer use and increased total sitting time [46]. While our study adjusted for participation in moderate/vigorous physical activity, this covariate did not encompass sedentary behaviors. There is also evidence of widening racial/ethnic and socio-economic disparities in obesity in recent years [47], which may not be sufficiently accounted for by adjustment for race/ethnicity and PIR.

Despite some of the associations attenuating over time, this study reaffirms that agricultural subsidies may play a role in diet-related risk factors for cardiometabolic disease. It is important to recognize the historical reasons behind subsidizing commodities; subsidies were initially implemented as a method of improving public health by ensuring a consistent food supply, as well as a way to support prices of foods and income for farmers [48]. However, the US produces more calories per capita than are needed according to nutritional recommendations, with >2500 daily calories available per person after loss adjustment in 2010 [49]. At the same time, there remains an insufficient supply of fruits and vegetables to meet nutritional recommendations, with an estimated 22% supply gap globally and a 19% supply gap in the US [50]. One strategy that may alleviate the rise in cardiometabolic diseases includes increasing the production of healthier foods, such as fruits and vegetables [51]. A recent study modeled the impact of price changes by using estimates of the average change in intake in response to a 1% change in price for each food group and examined the effect on mortality. They found that altering the price of seven dietary factors by 10% (decreasing the price of healthy dietary factors and increasing the price of unhealthy dietary factors) could prevent 23,000 deaths per year, representing 3.4% of all deaths associated with cardiometabolic disease in the US [12]. A similar model based on the Australian population examined the impact of a combined effect on taxing unhealthier foods (foods high in saturated fat, salt, sugar, and SSBs) and subsidizing healthier foods (fruits and vegetables). They found that the combined approach averted as many as 470,000 disability-adjusted life years, with cost-savings of AU$3.4 billion (~$2.3 billion) [52]. In a model examining the impact of a fruit and vegetable subsidy on the health-adjusted life years of New Zealand’s population, 212 health-adjusted life years were gained through a 20% subsidy on fruits and vegetables [53]. These studies hint at the positive effect of redistributing agricultural subsidies to support the production of fruits and vegetables, in order to reduce cardiometabolic diseases.

This study has several limitations. We are limited by the cross-sectional nature of the data, so we cannot establish causality. However, these findings are consistent with previous results in a separate population, increasing our confidence in the results. Additionally, we used single 24-hour recalls to determine the subsidy score, which are prone to bias due to variability in the diet and the many known limitations of 24-hour recalls, including underreporting [54]. An important limitation is the temporality of the associations we measured. While our current and previous study are meant to examine the effects of specific Farm Bills (2002 and 2008) on the diet, it is difficult to estimate the time lag between the implementation of the policy to the actual production of food that would then be consumed and measured by NHANES. Therefore, the associations we identified may represent the effects of previous Farm Bill policies. Several limitations of the subsidy score have been reported previously [14]. These include the use of averages in the energy content per gram to produce the subsidy score, which may overestimate the subsidy score. Furthermore, we were not able to include by-products of subsidized commodities (such as soy lecithin) in this analysis. However, due to their small contribution to the diet, it is unlikely that they would change the results substantially. We are also unable to differentiate between foods derived from subsidized commodities grown in the U.S. and imported foods. Estimates from the USDA suggest that 18.3% of the food purchases are from imported foods, where 10% could be from foods that are subsidized in the US (including beef, pork, poultry and eggs, dairy, grains, and rice). However, the vast majority of this estimate is from rice (4.5%) [55]. Therefore, 10% of the subsidy score could be derived from imported foods; however, this is likely the upper estimate.

In summary, this study agrees with previous literature suggesting that a higher consumption of foods derived from subsidized commodities is associated with obesity, abdominal adiposity, and dysglycemia, and further reinforces the potential benefits of aligning agricultural policy with health recommendations.

## Figures and Tables

**Table 1:** Characteristics of US adults aged 18 to 64 years in the National Health and Nutrition Examination Survey from 2009–2014 overall and by subsidy score quartiles.

Variable	Unweighted Number	Weighted Distribution ^A^	Q1 (0.00–0.41) ^A^	Q2 (0.42–0.53) ^A^	Q3 (0.54–0.63) ^A^	Q4 (0.64–1.00) ^A^	*p*-Value ^B^
Subsidy Score, mean (95% CI)	12,039	0.50 (0.50–0.51)	0.30 (0.30–0.31)	0.47 (0.47–0.47)	0.58 (0.58–0.59)	0.72 (0.71–0.72)	
Male	6279	49.5 (0.5)	48.6 (0.9)	50.3 (1.0)	48.1 (1.4)	51.0 (1.1)	0.97
Age group, year							
18–24	2058	15.2 (0.8)	13.3 (1.1)	13.6 (1.0)	15.8 (1.6)	18.7 (1.0)	<0.0001
25–34	2360	20.7 (0.6)	19.6 (1.1)	21.3 (1.0)	20.4 (1.2)	21.8 (1.0)	
35–44	2445	21.0 (0.7)	21.5 (1.1)	20.3 (1.0)	21.4 (1.1)	21.0 (1.2)	
45–54	2498	22.8 (0.7)	24.0 (1.1)	22.7 (1.0)	23.5 (1.6)	20.6 (1.0)	
55–64	2403	20.3 (0.6)	21.7 (1.1)	22.1 (1.1)	18.9 (1.3)	17.8 (1.0)	
Age, mean (SE), year	12,039	40.9 (0.3)	41.9 (0.4)	41.6 (0.4)	40.6 (0.6)	39.4 (0.4)	<0.0001
Race/ethnicity							
Non-Hispanic white	4683	65.1 (2.1)	63.9 (2.5)	67.2 (2.3)	65.6 (2.5)	63.9 (2.6)	0.41
Non-Hispanic black	2549	11.5 (1.0)	11.8 (1.3)	11.3 (1.2)	11.2 (1.1)	12.0 (1.0)	
Mexican American	1884	9.6 (1.2)	8.9 (1.3)	9.0 (1.0)	9.8 (1.4)	10.8 (1.7)	
Other	2648	13.8 (0.8)	15.4 (1.2)	12.6 (1.0)	13.3 (1.2)	13.5 (1.3)	
Education Attainment							
<High school graduate	2716	15.9 (0.8)	12.8 (1.1)	15.2 (1.2)	16.1 (1.1)	20.5 (1.1)	<0.0001
High school graduate	2735	21.6 (0.7)	19.4 (1.2)	19.4 (0.8)	24.3 (1.5)	24.4 (1.2)	
Some college	3737	32.2 (0.8)	33.1 (1.3)	31.0 (1.2)	31.6 (1.5)	33.1 (1.1)	
≥College graduate	2839	30.3 (1.2)	34.7 (1.8)	34.5 (1.3)	28.1 (1.8)	22.0 (1.5)	
Poverty Income Ratio, % ^C^							
<130	3883	24.1 (1.2)	21.0 (1.5)	22.4 (1.4)	25.0 (1.6)	29.2 (1.6)	<0.0001
130 to <185	1311	9.9 (0.5)	9.0 (0.7)	10.0 (0.7)	10.0 (0.8)	11.1 (0.9)	
≥185	5647	65.9 (1.4)	70.0 (1.7)	67.6 (1.6)	65.0 (1.6)	59.7 (2.0)	
Smoking status							
Current	2648	22.0 (0.7)	20.8 (1.0)	21.1 (1.0)	22.7 (1.3)	24.2 (1.5)	0.70
Past	2074	20.3 (0.8)	22.7 (1.0)	20.4 (1.0)	19.1 (1.5)	18.2 (1.1)	
Never	6524	57.6 (1.0)	56.5 (1.1)	58.5 (1.3)	58.2 (2.1)	57.6 (1.7)	
Daily energy, mean (SE), kcal	12,039	2233.05 (9.8)	2257.8 (20.0)	2265.8 (20.1)	2240.0 (25.1)	2159.6 (19.3)	0.0004
Leisure-time physical activity ^D^							
Yes	6223	57.3 (1.0)	62.1 (1.4)	57.6 (1.5)	56.9 (1.3)	51.2 (1.3)	<0.0001
No	5539	42.7 (1.0)	37.9 (1.4)	42.4 (1.5)	43.1 (1.3)	48.8 (1.3)	

^A^ Estimates are weighted percentages (standard errors (SE)), unless otherwise noted. Subsidy score quartiles were defined as follows: Q1 is 0.00–0.41; Q2 is 0.42–0.53; Q3 is 0.54–0.63; and Q4 is 0.64–1.00. ^B^ Differences in subsidy score quartiles with categorical variables examined via chi-square tests and with continuous variables examined via ANOVA. ^C^ Poverty income ratio defined by an individual’s eligibility for food assistance programs. ^D^ Level of physical activity was defined by individuals participating in at least 10 min of leisure-time moderate or vigorous physical activity daily in a typical week.

**Table 2:** Cardiometabolic risk factors by subsidy score quartiles, taken from the National Health and Nutrition Examination Survey from 2009–2014.

Variable	Overall Mean ^A^	Q1 (0.00–0.41) ^A^	Q2 (0.42–0.53) ^A^	Q3 (0.54–0.63) ^A^	Q4 (0.64–1.00) ^A^	*p*-Value ^B^
Body mass index (kg/m^2^)	28.8 (28.5, 29.0)	28.5 (28.1, 28.9)	28.8 (28.5, 29.1)	29.0 (28.6, 29.4)	29.4 (29.0, 29.8)	0.003
Ratio of waist circumference to height	0.578 (0.574–0.582)	0.575 (0.569–0.580)	0.576 (0.571–0.580)	0.584 (0.578–0.590)	0.586 (0.581–0.592)	<0.0001
Systolic blood pressure, mm Hg	118.7 (118.2–119.2)	118.8 (118.0–119.6)	118.9 (118.3–119.5)	119.1 (118.2–120.0)	118.8 (117.9–119.6)	0.90
Diastolic blood pressure, mm Hg	71.2 (70.6–71.8)	71.2 (70.6–71.8)	71.4 (70.7–71.1)	71.5 (70.7–72.2)	71.9 (70.9–72.9)	0.60
Non-HDL cholesterol concentration, mg/dL	140.7 (139.4, 142.0)	139.4 (137.8, 140.9)	140.8 (138.6, 142.9)	141.8 (139.8, 143.7)	143.8 (141.6, 146.1)	0.004
Hemoglobin A1c level, %	5.54 (5.52–5.56)	5.51 (5.47, 5.54)	5.54 (5.50, 5.58)	5.56 (5.52, 5.60)	5.58 (5.54, 5.62)	0.037

^A^ Estimates are adjusted means (95% confidence interval) calculated from multivariable linear regression, controlling for sex, age, race/ethnicity, the highest education level, the poverty–income ratio, the smoking status, and participation in moderate to vigorous physical activity, with missing data excluded from the model. Subsidy score quartiles were defined as follows: Q1 is 0.00–0.41; Q2 is 0.42–0.53; Q3 is 0.54–0.63; and Q4 is 0.64–1.00. ^B^ Differences in mean cardiometabolic risk factors by quartile were examined using multivariable linear regression.

**Table 3:** Cardiometabolic risk factors by continuous subsidy score, obtained from the National Health and Nutrition Examination Survey from 2009–2014.

Variable	Coefficient (Standard Error) ^A^	*p*-Value ^B^
Body mass index (kg/m^2^)	0.23 (0.05)	<0.0001
Ratio of waist circumference to height	0.003 (0.001)	<0.0001
Systolic blood pressure, mm Hg	0.05 (0.001)	0.68
Diastolic blood pressure, mm Hg	0.17 (0.11)	0.11
Non-HDL cholesterol concentration, mg/dL	1.11 (0.26)	<0.0001
Hemoglobin A1c level, %	0.02 (0.006)	<0.0001

^A^ Estimates denote the change in cardiometabolic risk factor per tenth of a point increase in subsidy score controlling for sex, age, race/ethnicity, the highest education level, the poverty–income ratio, the smoking status, and participation in moderate to vigorous physical activity, with missing data excluded from model. ^B^ Differences in mean cardiometabolic risk factors by the continuous subsidy score were examined using multivariable linear regression.

**Table 4:** Changes in food components making up the subsidy score.

Food Component	Mean (SE) 2001–2006	Mean (SE) 2009–2014	Relative Change	*p*-Value
Corn Sweetener	0.085 (0.002)	0.075 (0.001)	12%	<0.0001
Dairy	0.117 (0.002)	0.100 (0.001)	14%	<0.0001
Grains	0.223 (0.001)	0.201 (0.001)	10%	<0.0001
Soy	0.048 (0.005)	0.042 (0.004)	14%	0.002
Eggs	0.015 (0.0004)	0.008 (0.0002)	51%	<0.0001
Meat	0.133 (0.001)	0.129 (0.002)	2%	0.21
Corn-fed Fish	0.00006 (0.00005)	0.00005 (0.0004)	32%	0.15

Changes in mean food components making up the subsidy score in the National Health and Nutrition Examination Survey between 2001–2006 and 2009–2014. Food components are categories used to derive the subsidy score. Estimates are adjusted means (SE) calculated from multivariable linear regression, controlling for sex, age, race/ethnicity, the highest education level, the poverty–income ratio, the smoking status, participation in moderate to vigorous physical activity, and total caloric intake, with missing data excluded from the model.

## Supplementary Materials

The following are available online at https://www.mdpi.com/2072-6643/12/11/3244/s1: Figure S1: Weighted distribution of subsidy score. Figure S2: Association between continuous subsidy score and BMI (2A), waist to height ratio (2B), non-HDL cholesterol (2C) and hemoglobin A1c (2D); Table S1. Cardiometabolic risk factors by subsidy score quartiles, National Health and Nutrition Examination Survey from 2009-2014; Table S2: Adjusted prevalence ratio of cardiometabolic risk factors by subsidy score quartiles, National Health and Nutrition Examination Survey from 2009-2014.
