# Supplementary material for: Consumption of Foods Derived from Subsidized Crops Remains Associated with Cardiometabolic Risk: An Update on the Evidence Using the National Health and Nutrition Examination Survey 2009–2014
Source: Nutrients. 2020 Oct 23;12(11):3244. doi: 10.3390/nu12113244 (PMC7690710; doi:10.3390/nu12113244)
Supplement: Supplementary file 1 [file nutrients-12-03244-s001.pdf]

Supplemental Tables and Figures

Supplemental Figure S1

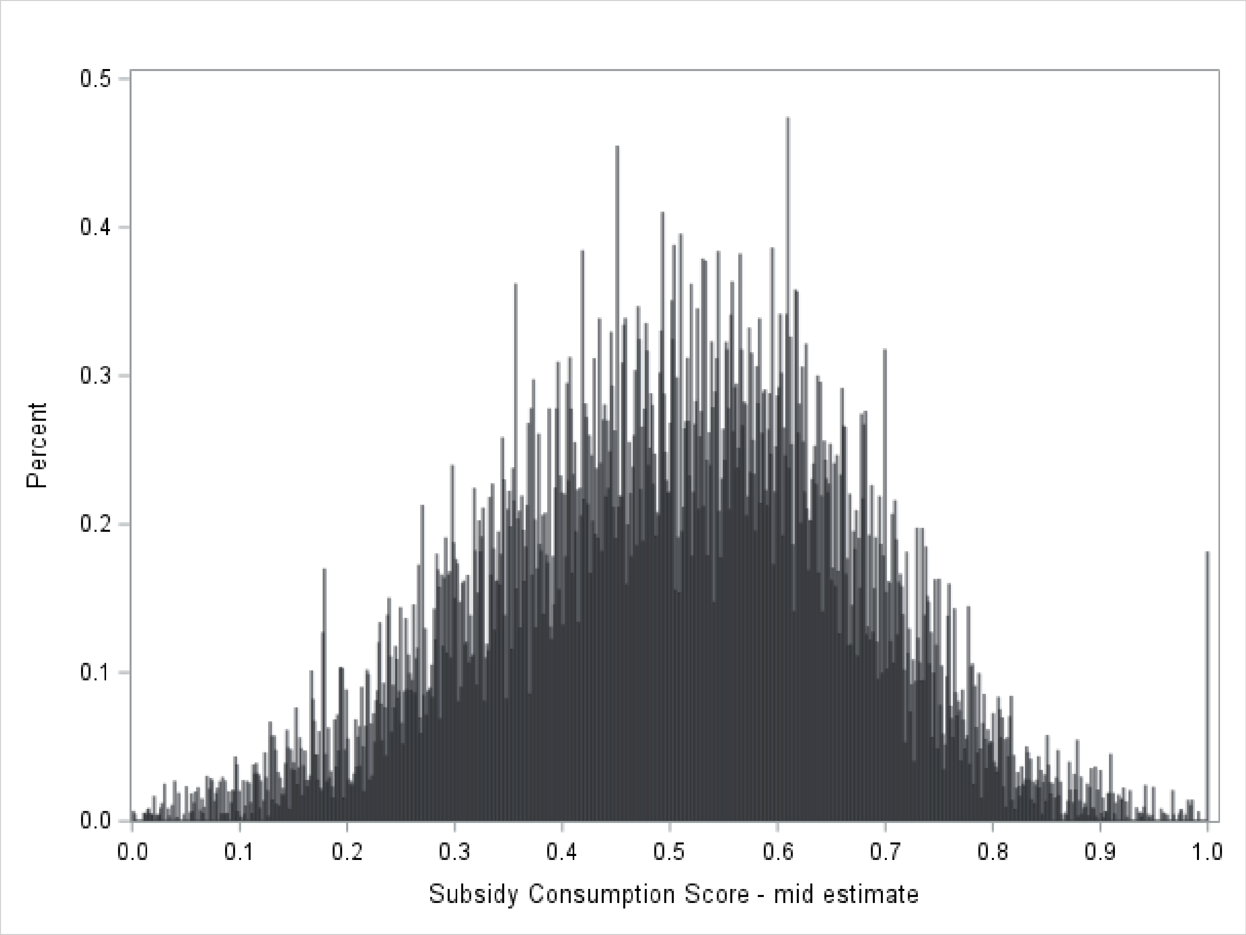

Supplemental Figure S1. Weighted distribution of subsidy score.

Supplemental Figure S2

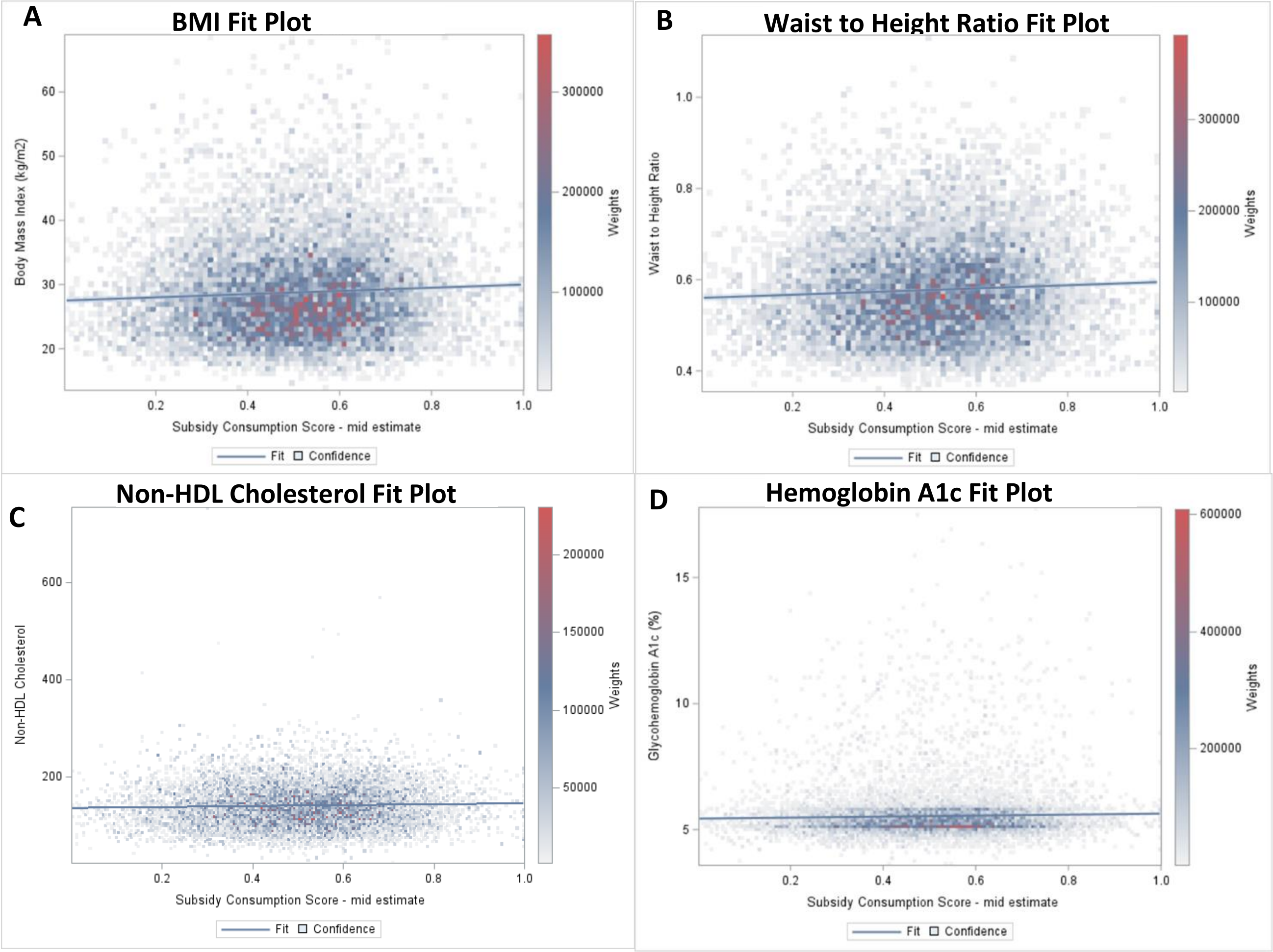

Supplemental Figure S2. Association between continuous subsidy score and BMI (2A), waist to height ratio (2B), non-HDL cholesterol (2C) and hemoglobin A1c (2D).

Supplemental Table S1. Cardiometabolic risk factors by subsidy score quartiles, National Health and Nutrition Examination Survey from 2009-2014

Variable	Overall Mean	Q1	Q2	Q3	Q4	p-value
Body Mass Index (kg/m^2)	28.8 (28.6, 29.1)	28.6 (28.3, 28.9)	29.0 (28.6, 29.3)	29.3 (28.8, 29.8)	29.4 (29.0, 29.8)	0.0015
Ratio of waist circumference to height	0.578 (0.574-0.582)	0.572 (0.567-0.577)	0.578 (0.573-0.583)	0.586 (0.578-0.593)	0.583 (0.577-0.589)	0.0002
SBP, mm Hg	118.8 (118.2-119.3)	118.8 (118.2 -119.4)	119.57(119.0-120.5)	118.4 (117.5-119.4)	119.1 (118.1-120.1)	0.052
DBP, mm Hg	71.3 (70.7-71.8)	71.3 (70.8-71.9)	71.8 (71.1-72.6)	71.7 (70.7-72.6)	72.1 (71.1 -73.1)	0.40
Non-HDL cholesterol level, mg/dL	140.8 (139.5, 142.0)	140.5 (138.8, 142.1)	140.3 (138.0, 142.6)	143.8 (141.5, 146.1)	144.9 (142.4, 147.4)	0.0017
Glycated hemoglobin level, %	5.54 (5.52-5.56)	5.52 (5.48, 5.55)	5.55 (5.51, 5.59)	5.58 (5.53, 5.63)	5.60 (5.55, 5.64)	0.01

Supplemental Table S1. Subsidy score quartiles were defined as follows using cutoffs from previous analysis of 2001-2006 data: Q1 is 0.00-0.47, Q2 is 0.48-0.57, Q3 is 0.58-0.65, and Q4 is 0.66-1. Adjusted for sex, age, race/ethnicity, highest education level, poverty-income ratio, smoking status, participation in moderate to vigorous physical activity and total daily caloric intake with missing data excluded from model.

Supplemental Table S2. Adjusted prevalence ratio of cardiometabolic risk factors by subsidy score quartiles, National Health and Nutrition Examination Survey from 2009-2014

Subsidy Score	Obesity	Abdominal Adiposity	Hypertension	Dyslipidemia	Dysglycemia
Q1	1	1	1	1	1
Q2	**1.17 (1.00, 1.36)**	1.10 (0.95, 1.27)	1.10 (0.95, 1.27)	0.86 (0.73, 1.01)	1.09 (0.95, 1.26)
Q3	**1.22 (1.00, 1.48)**	**1.25 (1.06, 1.48)**	0.99 (0.84, 1.18)	1.09 (0.93, 1.28)	1.17 (0.95, 1.42)
Q4	**1.27 (1.09, 1.47)**	**1.19 (1.01, 1.41)**	1.08 (0.92, 1.26)	1.03 (0.86, 1.24)	**1.32 (1.13, 1.54)**

Supplemental Table S2. Subsidy score quartiles were defined as follows: Q1 is 0.00-0.47, Q2 is 0.48-0.57, Q3 is 0.58-0.65, and Q4 is 0.66-1.00. Obesity was defined as body mass index of at least 30 kg/m^2. Abdominal adiposity was defined as a ratio of waist circumference to height of at least 0.59. Hypertension was defined as diagnosed (self-reported) or undiagnosed (no self-reported diagnosis and SBP≥140 mm Hg or DBP≥90 mm Hg) or currently taking antihypertensive medication. Dyslipidemia was defined as diagnosed (self-reported) or undiagnosed (no self-reported diagnosis and non-HDL cholesterol level ≥160 mg/dL) dyslipidemia or currently taking anticholesterolemia medication. Dysglycemia was defined as self-reported diabetes diagnosis or hemoglobin A1c level of at least 5.7%. Individuals with missing data were excluded from the models. Model adjusted for sex, age, race/ethnicity, educational attainment, poverty income ratio, smoking status, moderate and vigorous leisure time physical activity and total caloric intake.
